# Supplementary material for: Expression Quantitative Trait Locus of Wood Formation-Related Genes in Salix suchowensis
Source: Int J Mol Sci. 2023 Dec 23;25(1):247. doi: 10.3390/ijms25010247 (PMC10778782; doi:10.3390/ijms25010247)
Supplement: Supplementary file 1 [file ijms-25-00247-s001.zip › Supplementary Figure S1.pdf]

A

Auxin

-1.68	*GH3.10*
3.51	*AUX28*
1.93	*IAA26*
-4.59	*SAUR21.1*
1.85	*SAUR21.2*
-2.77	*SAUR36*

JA

-1.60	*JAZ/TIFY 10A.1*
-1.63	*JAZ/TIFY 10A.2*
-2.18	*JAZ/TIFY 9.1*
-2.01	*JAZ/TIFY 9.2*
-2.62	*JAZ/TIFY 9.3*
-1.04	*MYC2*

Cytokinin

2.77	*ARR-A/ARR5*

Ethylene

-4.00	*ETR/EIN4*

ABA

1.21	*SnRK2/SAPK3*

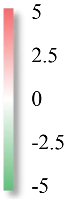

B

	t1	t2	t3	t4	t5	t6
BAM1	-0.19	-0.06	-0.41	-1.13	-0.22	-0.26
CDKB2;1	0.3	0.98	-1.49	-1.19	-0.04	-1.86
CLV1	-1.8	-1.81	2.21	-1.59	0.26	0.44
CYCB2;4	0.6	0.8	-1.46	-1.5	-0.22	-1.33
HAT22	-0.14	-0.17	0.33	0.96	-0.03	-0.19
JAZ1	-0.75	-0.26	-2.05	-0.43	NA	-3.12
MYB36	-1.85	0.12	0.23	0.88	1.38	-0.24

Supplementary Figure S1. Visualization of DEGs. **(A)** Log2 (fold change) of hormone-related genes in the parents (NF2 vs LS7). **(B)** Log2 (fold change) of candidate genes in two contrasting clones of *S. suchowensis* throughout the entire growth process.
