# Supplementary material for: Expression Quantitative Trait Locus of Wood Formation-Related Genes in Salix suchowensis
Source: Int J Mol Sci. 2023 Dec 23;25(1):247. doi: 10.3390/ijms25010247 (PMC10778782; doi:10.3390/ijms25010247)
Supplement: Supplementary file 1 [file ijms-25-00247-s001.zip › Supplementary Figure S2.pdf]

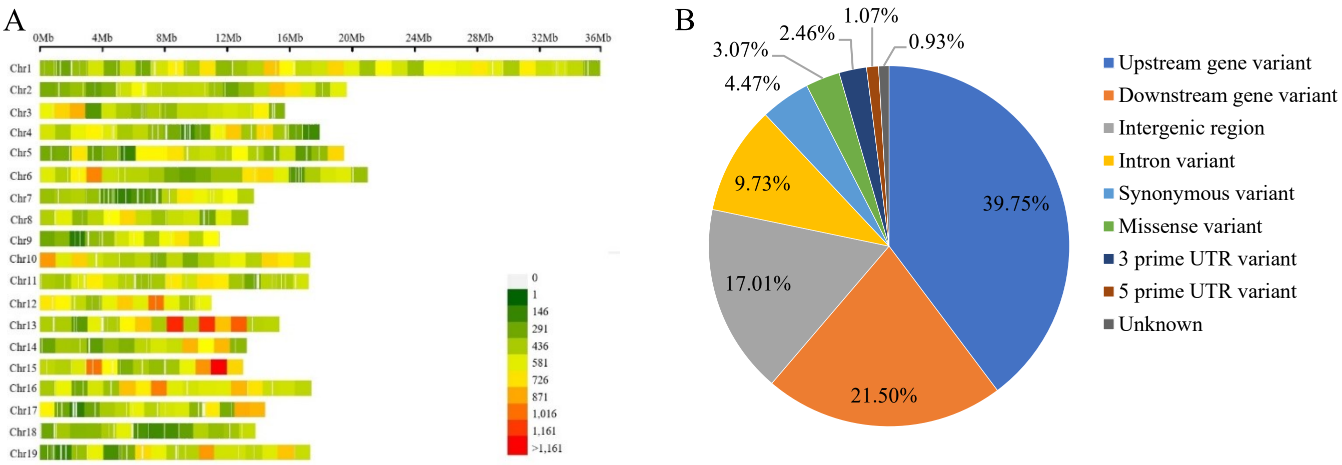

Fingure S2. Distribution of SNPs in the *S. suchowensis* genome. (**A)** Visualization of SNP density across 19 chromosomes. (**B)** Counts of SNPs for different types of variations.
