# Supplementary figures and images for: Expression Quantitative Trait Locus of Wood Formation-Related Genes in Salix suchowensis
Source: Int J Mol Sci. 2023 Dec 23;25(1):247. doi: 10.3390/ijms25010247 (PMC10778782; doi:10.3390/ijms25010247)

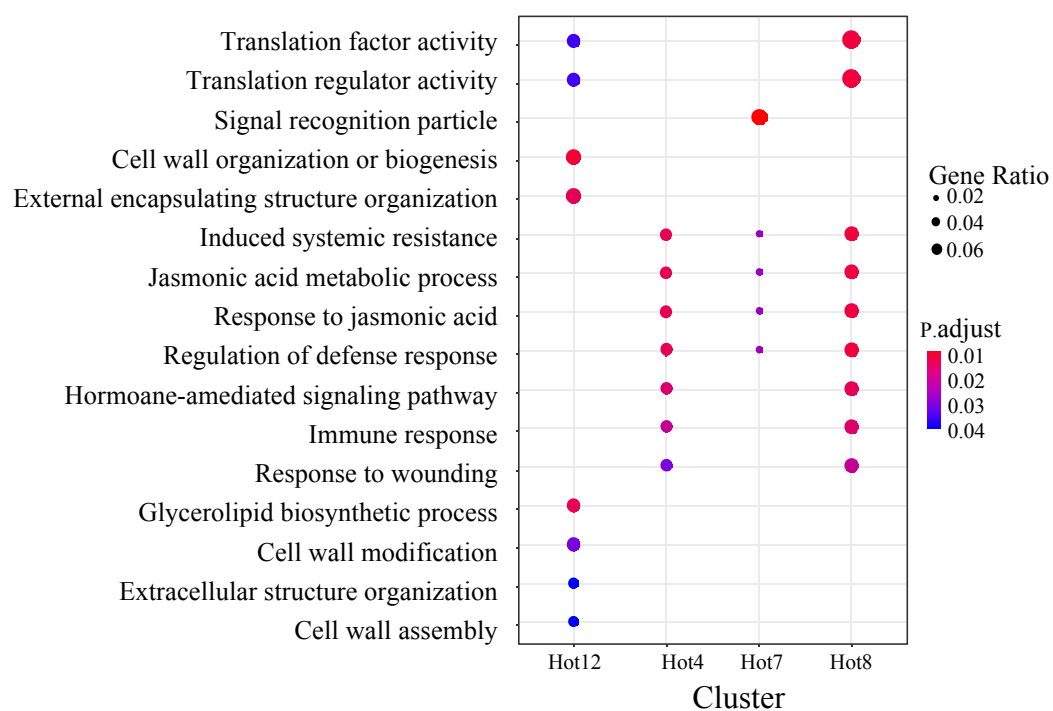

**Supplementary Figure S3.** GO enrichment analysis of targets regulated by 4 hotspots.

Supplement: Supplementary file 1 [file ijms-25-00247-s001.zip › Supplementary Figure S3.pdf]
